# Contour Filling

**Published:** 1874-03

**Authors:** G. W. Field

**Affiliations:** Geneva, Switzerland


### CONTOUR FILLING.

BY DR. G. W. FIELD, GENEVA, SWITZERLAND.

*Read before the American Dental Society of Europe.*

While reading, recently, an Essay on Contour Filling, written for and read before an American Dental Society, I could but draw the contrast in my mind between the European and American demands and practice.

If in the discharge of the professional duties of an American practice there are discouragements to be met and contended against, and obstacles to be overcome; if where the people in general are better informed, and more intelligent on the subject of the care of the dental organs, and as a consequence more appreciative of our efforts to preserve them; if in the midst of such enlightenment there are difficulties in the way of such conservative practice as the essayist just alluded to advocates, what would he and our fellow operators in general of America say to the blackness of darkness in which we here labor. When our patients prefer to lose their teeth, or wear a black unsightly mastic until it drops out—tooth and all, rather than submit to a little pain, a long operation or pay more than day laborer's wages for the most arduous operations; and if, perchance, we succeed in making the operation and having expended much time, patience and skill in producing such results as shall please the patient and gratify our own pride, we give him a mirror to examine our beautiful filling, and to express his admiration, but instead of that he disgusts us by exclaiming with eyes and mouth staring, "how dear." One sees the gold. Two hours previous to this he was content to see and show an amalgam filling of the blackest and encircled by decay; now he objects to a clean tooth because one sees pure gold. Our American brother might well call these discouragements, by the side of which, their own would vanish. Notwithstanding the many objections which may be urged by both patient and dentist, my voice is still for contour fillings, even to the building up of a whole crown, provided the roots are firm and healthy.

The teeth were designed for use, pre-eminently so, in fact, I doubt if our creator thought of ornament, but rather of adapting means to the end. All things were in readiness and awaiting the advent of man; the Almighty then made him and adapted his organism to the food already prepared, and as stoves and cooking utensils were of a later invention, I infer that the teeth were more useful than ornamental. Admitting that the dental organs are for use, we must then admit that we fall far short of our duty towards our patients if we do not our utmost to preserve the utility of defective organs.

Let us take nature as our guide, and contour fillings will need no words of recommend from such a pen as mine. He does not give us inclined planes upon which to masticate our food, and prepare it for that delicate organ the stomach, nor saw teeth with which to cut our morsels, but thin square edges for the one and broad indented surfaces for the other.

The form of the front teeth have much to do, as has also the molars, with the articulation of words, as well as being important to preserve the facial expression. How often have we heard the whistling or whirring sound resulting from too much space between the front teeth, and have seen the tongue spread to nearly double its usual width, and the patient talking with some difficulty on account of the loss of the molars and bicuspids. If it is once more brought under subjection and confined within its natural limits by an artificial denture, the patient informs us that she can speak with greater ease than for years previous. How many will deny these facts ? and I deem of great weight in any argument to prove the utility of contour fillings.

I would build out an incisor, canine or bicuspid one-half, rather than make a flat filling, and invariably so if the pulp was not exposed.

In the case of central incisor and which is badly decayed and broken, leaving but about two-thirds or, perhaps, only one-half the crown intact; I would remove all decay and cut away all softened dentine, and frail or suspicious enamel, and wherever possible would make a slight under cut. Instead of depending upon retaining points for the security and solidity of my fillings, I would insert two gold screws, which, by the way, are not Mack's, but like watch-maker's screws. One of these would be inserted to lower edge of pulp cavity and cutting-edge of tooth, and at a right angle with the tooth. The second would be screwed into the dentine; the thick portions by the side of the pulp cavity, and parallel with the tooth. The screws are mode of 12 K. gold. The thread is cut the whole length. There is a small head, just large enough to have a small slot to receive the miniature screw-driver. I use no taps to prepare the hole, but simply bore a hole a shade smaller than the screw and I find that they take a firm hold in the dentine, sufficient to support the largest fillings.

Before commencing the fillings, the edges of the cavity should be thoroughly finished to assure a perfect joint between the gold and enamel. I commence the introduction of the gold in a retaining point and from that build to and around the screw, always applying the force towards the center of the tooth. For gold, Abbey's adhesive, No. 4 is my main dependence, but prefer to finish with Williams' 30, 60 or 240. A filling made in this manner will finish without pits or prickles, and will stand to demonstrate by its usefulness the wisdom of your choice for contour fillings.

As to the unsightliness of such operations, it certainly is more suggestive of beauty than the spaces left by flat fillings. I have thus far only spoken of the anterior teeth, but those occupying a less conspicuous position, the molars, must not be over-looked, for they are of greater importance, as regards usefulness, than their forward sisters, for the latter can be substituted to very good advantage, but who of us will say that he can furnish a substitute for a noble molar. Yes, we do furnish substitutes, but many of them are bounty jumpers.

The molars and bicuspids also should be built contour. If we are not able to do it with gold, by reason of something constitutional, or other good reason; or the patient is not able to pay for such an operation, let us still say contour, and make use of the much abused amalgam, or even Gulloi's cement will answer a good purpose. During the past year, I saw a superior 1st molar about two-thirds contour of Gulloi's, it had then been in service over a year, and was still perfect. Will we presume to say that the inclined plan would have been better than this hard, solid, grinding surface.

One great objection raised is the unwillingness of the patient to pay a compensating fee for the time, labor and skill required to produce satisfactory operations. And there is an obstacle which rises like a mountain before us, for ours is a profession that taxes our every nerve and saps our life, and in very many instances for our best efforts we are not compensated. Still I think that this objection will gradually vanish. We can always find a class of people who demand the very best talent and greatest skill and are willing to give value received, not only in hard cash, but also that which is many times more than money, appreciation of our efforts to benefit them. I have operated for half fee, for intelligent, appreciative people of only moderate means, and I feel none the poorer. Perhaps it may require some effort on our part to present to the minds of our patients the advantages of their imitating nature, and if our words are wisely chosen and cautiously presented they will readily see that money is not our only object, but that we have a love for our profession which prompts us to aim at the highest possible perfction in all our operations and to seek the good of our patients, rather than be always influenced by the sordid love of gain.

